# VESTIGE: Adjuvant Immunotherapy in Patients With Resected Esophageal, Gastroesophageal Junction and Gastric Cancer Following Preoperative Chemotherapy With High Risk for Recurrence (N+ and/or R1): An Open Label Randomized Controlled Phase-2-Study

**DOI:** 10.3389/fonc.2019.01320

**Published:** 2020-01-30

**Authors:** Elizabeth Smyth, Maren Knödler, Anne Giraut, Murielle Mauer, Magnus Nilsson, Nicole Van Grieken, Anna Dorothea Wagner, Markus Moehler, Florian Lordick

**Affiliations:** ^1^Cambridge University Hospitals NHS Foundation Trust, Cambridge, United Kingdom; ^2^University Cancer Center Leipzig, University Hospital Leipzig, Leipzig, Germany; ^3^EORTC Headquarters, Brussels, Belgium; ^4^Division of Surgery, Department of Clinical Science, Intervention and Technology, Karolinska Institutet and Karolinska University Hospital, Stockholm, Sweden; ^5^Department of Pathology, Amsterdam UMC, Cancer Center Amsterdam, VU University, Amsterdam, Netherlands; ^6^Department of Oncology, Lausanne University Hospital and University of Lausanne, Lausanne, Switzerland; ^7^University Medical Center, Johannes Gutenberg University Mainz, Mainz, Germany

**Keywords:** gastric cancer, gastroesophageal cancer, immunotherapy, chemotherapy, adjuvant, nivolumab, ipilimumab, perioperative

## Abstract

**Background:** Perioperative chemotherapy plus surgery is one recommended standard treatment for patients with resectable gastric and esophageal cancer. Even with a multimodality treatment more than half of patients will relapse following surgical resection. Patients who have a poor response to neoadjuvant chemotherapy and have an incomplete (R1) resection or have metastatic lymph nodes in the resection specimen (N+) are especially at risk of recurrence. Current clinical practice is to continue with the same chemotherapy in the adjuvant setting as before surgery. In the phase II randomized EORTC VESTIGE trial (NCT03443856), patients with high risk resected gastric or esophageal adenocarcinoma will be randomized to either adjuvant chemotherapy (as before surgery) or to immunotherapy with nivolumab and low dose ipilimumab (nivolumab 3 mg/kg IV Q2W plus Ipilimumab 1 mg/kg IV Q6W for 1 year). The primary endpoint of the study is disease free survival, with secondary endpoints of overall survival, safety and toxicity, and quality of life. This is an open label randomized controlled multi-center phase-2 superiority trial. Patients will be randomized in a 1:1 ratio to study arms. The trial will recruit 240 patients; recruitment commenced July 2019 and is anticipated to take 30 months. Detailed inclusion/exclusion criteria, toxicity management guidelines, and statistical plans for EORTC VESTIGE are described in the manuscript.

**Clinical Trial Registration:** The trial is registered with www.ClinicalTrials.gov, identifier: NCT03443856.

## Introduction

### Background and Rationale

Gastric and esophageal cancer are amongst the most prevalent cancers globally; gastric cancer was diagnosed in 1,033,701 patients worldwide in 2018, whereas esophageal cancer occurred in 572,034 cases globally (1). Both gastric cancer and esophageal adenocarcinomas are associated with poor survival for patients with metastatic disease. Median survival is <1 year, and even for patients with resectable cancers who are treated with optimum multimodality treatment and surgery, long term survival is <50% (2–4). Perioperative chemotherapy is one standard of care in Europe for treatment of AJCC 8th edition clinical stage Ib—IVa (resectable) gastric and esophagogastric junction (EGJ) adenocarcinoma, according to the current European Society for Medical Oncology (ESMO) clinical practice guidelines; in non-Asian countries for esophageal and junctional adenocarcinoma chemoradiotherapy may also be considered whereas in Asia, adjuvant chemotherapy is preferred for gastric cancers (5, 6). Compared with resection alone, this treatment has increased survival rates by about up to 15% after 5 years of follow-up (7, 8). Appropriate chemotherapy regimens include platinum (either oxaliplatin or cisplatin) and fluoropyrimidine doublets or triplet chemotherapy. Since 2017, the FLOT regimen (docetaxel, oxaliplatin, fluorouracil, and leucovorin) has been considered the treatment of choice for patients who are fit for three drug combinations (4).

### Negative Prognostic Features in Patients Treated With Perioperative Chemotherapy and Surgery

In the MAGIC randomized control trial which defined perioperative ECX (epirubicin, cisplatin, and capecitabine) chemotherapy as a standard of care, patients who did not achieve a good pathological response to preoperative chemotherapy and who present with a positive nodal status (ypN+) or with an R1 resection status after preoperative chemotherapy have a very poor prognosis (9, 10). In this study the median survival of patients with node positive resected cancer (ypN1-3) was only 16 months and the 5-year survival rate was only 20%. It is notable that most recurrences occur early, within the first 2 years of follow-up. The prognosis of good responders to preoperative chemotherapy, who have a negative nodal status and undergo R0 resection, in contrast, is better. The median overall survival for all node-negative patients (regardless of the pathological tumor regression status) was not reached because it was greater than the longest censoring time. The 5-year survival rate was 66 and 71% for nodal-negative responders and non-responders; negative nodal status being the only independent positive prognostic factor in multivariate analysis (5).

Currently, patients who have a poor response to chemotherapy (due to remaining positive lymph nodes or a R1 resection) continue with the same treatment postoperatively as they did preoperatively as all the trials which defined these treatment approaches used pre and post-operative chemotherapy. However, within the oncology community there is a desire for better treatments rather than continuing with the same treatment which has been less effective than is desirable before surgery. Considering the known toxicities of classical chemotherapy regimens, a “switch” to a different treatment is attractive for high risk postoperative patients, but this should be assessed in the context of a randomization against the current recommended standard treatment which is postoperative adjuvant chemotherapy similar to that which the patient received before surgery.

Although survival for patients who have node positive disease after surgery in the FLOT4/AIO trial has not been presented, 51% of patients treated with FLOT were node positive (N+) after surgery, and 16% of patients had an R1 resection. Therefore, the proportion of patients who are high risk for recurrence after surgery is still high even in patients in whom FLOT is adopted as a standard of care (4). In general, therefore considering the activity of currently available chemotherapy regimens, the group of high risk non-chemoresponsive patients constitutes ~2/3 of those who undergo neoadjuvant chemotherapy. This relatively large group of patients has clear need for improved outcomes and better postoperative treatment.

### Immunotherapy for Gastric and Esophageal Adenocarcinoma: Rationale for PD-1 Inhibition

Immunotherapy in the form of checkpoint inhibition has shown efficacy in the treatment of patients with advanced gastric and esophagogastric junction (EGJ) cancers. Nivolumab is a humanized monoclonal, immunoglobulin G4 antibody directed against PD-1 which is licensed to treat melanoma, non-small cell lung cancer (NSCLC) and other cancers including small cell lung cancer, renal cell carcinoma, classical Hodgkin lymphoma, squamous cell carcinoma of the head and neck, urothelial cancer, MSI-H or dMMR metastatic colorectal cancer, and hepatocellular cancer (11–13).

Recently, nivolumab has received approval for advanced gastric cancer in Japan. In the ONO-4538-12 (ATTRACTION-2) trial, a phase III randomized study, patients with chemorefractory unresectable advanced or recurrent gastroesophageal cancer were randomized to either nivolumab 3 mg/kg every 2 weeks or placebo (14). The primary endpoint of the trial was overall survival, with secondary endpoints of progression free survival, best overall response and safety. A total of 493 patients were recruited and randomized in a 2:1 ratio to receive nivolumab or placebo. Treatment with nivolumab significantly improved median overall survival from 4.14 to 5.32 months [HR 0.63; 95% CI (0.50–0.78), *p* < 0.0001]. Survival at 12 months was almost doubled for nivolumab treated patients; this being 26.6% for nivolumab and 10.9% for patients treated with placebo. In nivolumab treated patients, RECIST responses were observed in 12% of patients, however some tumor shrinkage was observed in 40% of patients. A survival benefit for nivolumab treatment was observed for patients with and without PD-L1 expression (PD-L1 negative median OS 6.1 vs. 4.2 months nivolumab vs. placebo; PD-L1 positive median OS 5.2 vs. 3.8 months nivolumab vs. placebo) (15). Therefore, nivolumab is effective in chemorefractory gastric cancer regardless of PD-L1 status. Results like those observed in Asian patients with single agent nivolumab in ATTRACTION-2 have been demonstrated also in non-Asian patients in the CHECKMATE-032 study (16).

Similar results to nivolumab in gastric and gastroesophageal cancer have been demonstrated for pembrolizumab, which is a humanized immunoglobulin G4 monoclonal antibody targeting PD-1 which is licensed to treat melanoma, NSCLC and microsatellite unstable cancers of any tumor site (17–19). In the KEYNOTE 059 study cohort 1, 259 patients who had previously been treated with two or more lines of chemotherapy received pembrolizumab 200 mg Q3W (20). Approximately half (52%) of patients had tumors of the gastroesophageal junction. In KEYNOTE-059 cohort 1 radiological responses were observed in 12% of all patients (with a higher response rate in PD-L1 positive tumors [combined proportion score of ≥1] of 15%) and 42% of patients had some evidence of tumor shrinkage. These results are very consistent with those observed for single agent nivolumab, indicating a class effect of anti-PD-1 therapy in gastric and gastroesophageal cancer.

### Rationale for Combination CTLA4 and PD-1 Inhibition

Metastatic melanoma treated with combination anti-cytotoxic T lymphocyte associated protein 4 (CTLA4) and PD-1 therapy leads to increased response rates and progression free survival compared to single agent immunotherapy in particular for PD-L1 negative patients (12, 21–25). As most patients with gastroesophageal cancer have PD-L1 negative tumors, combination immune checkpoint blockade may be helpful in this disease. In two of the three of the Phase I/II CHECKMATE 032 study arms, nivolumab and ipilimumab were assessed at two dose levels; these were nivolumab 3 mg/kg plus ipilimumab 1 mg/kg Q3W (N3 plus I1) or nivolumab 1 mg/kg plus ipilimumab 3 mg/kg Q3W (N1 plus 13) (16). Landmark eighteen-month survival was 28% for N1 plus I3 patients and 13% for N3 plus I1 patients and radiological response rates were also increased for combination therapy, in particular for PD-L1 negative patients. Therefore, in a subsequent ongoing randomized trial in first-line stage IV gastric cancer, the N1 plus I3 regimen was selected for further investigation. However, this combination was found to result in excessive toxicity and a reduced dose of ipilimumab has been recommended as an alternative regimen depending on the setting.

Most recently, data on the safety and efficacy of dosing nivolumab at 3 mg/kg Q2W + 1 mg/kg Q6W dosing of ipilimumab has emerged from several trials (26). This dose was optimized across 8 cohorts of NSCLC in the Checkmate 012 study and then evaluated prospectively in the randomized Checkmate 227 study (27). In Checkmate 227 toxicity for the combination of nivolumab with low dose ipilimumab was similar compared with chemotherapy with respect to grade 3–4 treatment related adverse events. For example, the proportion requiring systemic corticosteroid use for immune related toxicity for each of the following organ systems was 14% (dermatological), 17% (endocrine), 36% (gastrointestinal), 44% (hepatic), 23% (renal), most of which resolved with steroid treatment. The combination of nivolumab 3 mg/kg Q2W and ipilimumab 1 mg/kg Q6W has now been evaluated in 941 patients across 3 trials (Checkmate 012, 227, and 568 studies) and found to have manageable toxicity with a low incidence of treatment-related adverse events leading to treatment discontinuation of between 12 and 17.4% in these studies. This represents a significant improvement on prior dosing schedules and therefore this regimen has been adopted for the VESTIGE trial.

## VESTIGE Study Objectives

### Primary Objective

The primary objective of the trial is to investigate if nivolumab plus ipilimumab given as adjuvant treatment improve disease free survival (DFS) in patients with AJCC 8th edition stage Ib-IVa gastric and esophagogastric (EG) junctional adenocarcinoma and high risk of recurrence (defined by ypN1-3 and/or R1 status) following neoadjuvant chemotherapy and resection.

### Secondary Study Objectives

To investigate the safety and effect of adjuvant immunotherapy on long term oncologic outcomes and quality of life of patients in the study.

To correlate nutritional status assessment with outcomes and quality of life of patients.

### Trial Design

This is an open label randomized controlled multi-center phase-2 superiority trial. Patients will be randomized in a 1:1 ratio to study arms.

**Figure:**
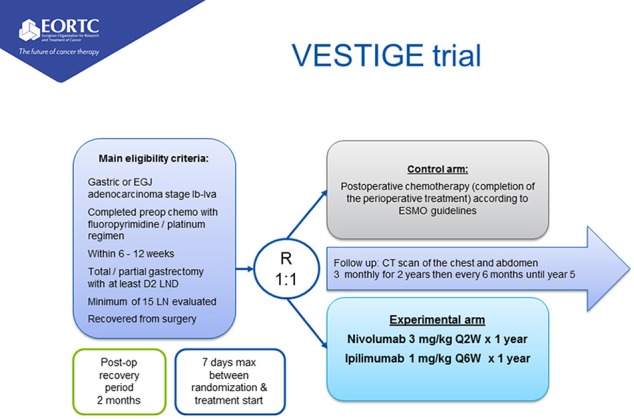


### Study Setting

The study will be conducted at EORTC study sites which may include academic and non-academic hospitals and cancer centers. The trial will be conducted in the following countries (Czech Republic, France, Germany, Israel, Italy, Norway, Poland, Portugal, Spain, United Kingdom). A list of sites can be found on clinicaltrials.gov.

### Eligibility Criteria

#### Inclusion Criteria

- Histologically proven gastric, lower esophageal or EG-junction adenocarcinoma (Siewert I-III).
- Subjects must have completed pre-operative chemotherapy with a fluoropyrimidine-platinum containing regimen and macroscopically complete surgery prior to randomization.
- Minimal duration of neoadjuvant chemotherapy should be 6 weeks, maximum 12 weeks.
- Total or distal gastrectomy with D2 lymphadenectomy according to ESMO guidelines should have been completed for gastric and EG junctional Siewert type III cancers.
- Ivor Lewis or McKeown esophagectomy with two field lymphadenectomy should have been performed for EG junctional Siewert type I cancers and lower esophageal adenocarcinomas.
- For Siewert type II cancers either total gastrectomy with D2-lymphadenectomy or esophagectomy with two field lymphadenectomy should have been completed.
- Open, minimal invasive or hybrid surgical approaches are acceptable as long as the requirements above are fulfilled.
- Regardless of the type of surgery a minimum of 15 lymph nodes should have been resected and examined.
- Recovered from surgery and fit for study treatment as assessed by a multidisciplinary team.
- Ideally, surgery should have been completed no longer than 2 months before randomization.
- ypN1-3 status according to current (8th) version of TNM classification system. In case of an ypN0 status patients must meet the inclusion criterion of R1 resection.
- R0 or R1 resection according to current (8th) version of TNM classification system. In case of R0 resection, patients must meet the inclusion criterion of ypN1-3.
- WHO performance status score of 0 or 1.
- Age ≥ 18 year.
- Adequate organ function assessed within 7 days before randomization:
  ° White blood cell count (WBC) > 2 × 10^9^/L.
  ° Absolute neutrophil count (ANC) > 1.5 × 10^9^/L.
  ° Platelets ≥ 100 × 10^9^/L.
  ° Hemoglobin ≥ 9 g/Dl.
  ° Measured/calculated creatinine clearance ≥ 60 mL/min (according to Cockroft-Gault formula).
  ° Total bilirubin within normal limits (if the patient has documented Gilbert's disease ≤ 1.5 ^*^ ULN or direct bilirubin ≤ ULN).
  ° Aspartate transaminase (AST) and alanine transaminase (ALT) ≤ 1.5ULN.
- Normal 12-lead ECG, and if clinically indicated, normal cardiac function assessment (using either echocardiography or MUGA Scan).
- In the past 6 months no serious cardiac illness or medical condition including but not confined to:
  ° History of documented congestive heart failure (CHF).
  ° High-risk uncontrolled arrhythmias.
  ° Angina pectoris requiring antianginal medication.
  ° Clinically significant valvular heart disease.
  ° Evidence of transmural infarction.
  ° Poorly controlled hypertension (e.g., systolic >180 mm Hg or diastolic >100 mm Hg).
- All toxicities (exception alopecia, and grade 2 fatigue, neuropathy, and lack of appetite /nausea) attributed to prior anti-cancer therapy must have resolved to grade 1 (NCI CTCAE version 5.0 or baseline before administration of study drug.
- Availability of resected tumor for biobanking/TR.
- Women of childbearing potential (WOCBP^*^) must have a negative serum or urine pregnancy test [minimum sensitivity 25 IU/L or equivalent units of human chorionic gonadotropin (HCG)] within 24 h prior to randomization.
- WOCBP should use highly effective method of birth control measures during the study treatment period and for at least 6 months after the last dose of the study treatment.
- Female subjects who are breast feeding should discontinue nursing prior to the first dose of study treatment and until 6 months after the last dose of the study treatment.
- Men who are sexually active with an WOCBP must adhere to contraception for a period of 7 months after the last dose of the study treatment.
- Absence of any psychological, familial, sociological, or geographical condition potentially hampering compliance with the study protocol and follow-up schedule; those conditions should be discussed with the patient before registration in the trial.
- Before patient registration/randomization, written informed consent must be given according to ICH/GCP, and national/local regulations.

### Exclusion Criteria

- R2 Resection Status.
- M1 stage according to the current (8th) version of TNM classification system.
- Patients who have undergone complete resection of metastases.
- Impaired renal, hepatic, cardiac, pulmonary, or endocrine status that compromises the eligibility of the patient for postoperative chemotherapy or immunotherapy.
- Subjects with previous malignancies are excluded unless a complete remission was achieved at least 5 years prior to study entry.
  ° Adequately treated cervical carcinoma *in situ*, and localized non-melanoma skin cancer are no exclusion criteria, regardless of timepoint of diagnosis.
- Subjects with active, known, or suspected infectious or autoimmune disease.
- Patients who have received antibiotics within the last 14 days before randomization are excluded.
- Subjects with Type I diabetes mellitus, residual hypothyroidism due to autoimmune thyroiditis only requiring hormone replacement, skin disorders (such as vitiligo, psoriasis, or alopecia) not requiring systemic treatment are permitted to enroll.
- Subjects with a condition requiring systemic treatment with either corticosteroids (≥ 10 mg daily prednisone or equivalent) or other immunosuppressive medications within 14 days of study drug administration.
- Subjects with interstitial lung disease who have clinical findings that may interfere with the detection or management of suspected drug-related pulmonary toxicity from the study.
- Subjects with > Grade 1 peripheral neuropathy.
- Prior treatment with an anti-PD-1, anti-PD-L1, anti-PD-L2, anti-CD137, or anti-CTLA-4 antibody, or any other antibody or drug specifically targeting T-cell co-stimulation or immune checkpoint pathways.
- Prior or concomitant treatment with radiotherapy/radiochemotherapy.
- Any positive test result for HBV or HCV indicating acute or chronic infection.
- Known history of testing positive for HIV or known AIDS.
- Known uncontrollable hypersensitivity to the components or excipients of nivolumab, ipilimumab, cisplatin/oxaliplatin, 5-FU or capecitabine, epirubicin or docetaxel.
- Known dihydropyrimidine dehydrogenase (DPD) deficiency.
- Ongoing or concomitant use of the antiviral drug sorivudine or its chemically related analogs, such as brivudine.

### Excluded Medications

The following medications are not permitted during the study (unless used to treat a drug related adverse event):

- Immunosuppressive drugs.
- Immunosuppressive doses of systemic corticosteroids (except for controlling immune related adverse events).
- Any other anti-cancer therapy (i.e., chemotherapy, hormonal therapy, immunotherapy, radiotherapy).
- Live vaccines < 4 weeks prior to and during study treatment are prohibited therapies. Examples of live vaccines include, but are not limited to, the following: measles, mumps, rubella, chicken pox, yellow fever, H1N1 flu, rabies, BCG, and typhoid vaccine.

The following medications should be avoided with capecitabine:

- Warfarin: patients receiving capecitabine and warfarin will require more frequent INR monitoring.
- Phenytoin: blood phenytoin levels can increase with capecitabine.
- Folic acid: multivitamin supplements containing folic acid should be avoided as it could potentially increase capecitabine toxicity.
- Allopurinol: potentially reduces the effectiveness of capecitabine.
- Interferon α: reduces maximal tolerated dose of capecitabine.

### Interventions

#### Experimental Arm

Total treatment time 1 year. No adjuvant chemotherapy. Treatment: nivolumab 3 mg/kg IV Q2W plus ipilimumab 1 mg/kg IV Q6W for 1 year.

#### Control Arm

Completion of the same perioperative treatment according to the 2016 ESMO guidelines (change of regimen is not allowed).

### Perioperative Chemotherapy May Include

^*^Physicians may use minor modifications of these regimens if this is judged acceptable by the local investigator.

#### Cisplatin + Fluorouracil (CF)

Fluouracil 800 mg/m^2^ IV continuous infusion over 24 h daily on Days 1–5; Cisplatin 75–80 mg/m^2^ IV on day 1; cycled every 28 days for 2–3 cycles preop and 3–4 cycles postop for a total of 6 cycles.

#### Cisplatin + Capecitabine (CX)

Cisplatin (80 mg/m^2^ every 3 weeks) and capecitabine (1,000 mg/m^2^ twice daily every 2 out of 3 weeks) for three cycles.

#### Epirubicin + Cisplatin + Fluorouracil (ECF)

Epirubicin 50 mg/m^2^ intravenously day 1, cisplatin 60 mg/m^2^ intravenously day 1, and 5-fluorouracil 200 mg/ m2/day intravenously continuous infusion over 24 h daily on days 1–21. Cycle is every 21 days for 3 cycles preop and post op.

#### Epirubicin + Cisplatin + Capecitabine (ECX)

Epirubicin 50 mg/m^2^ intravenously day 1, cisplatin 60 mg/m^2^ intravenously day 1, and capecitabine 625 mg/m^2^ twice daily on days 1–21. Cycled every 21 days for 3 cycles preop and 3 cycles postop.

#### Epirubicin + Oxaliplatin + Capectabine (EOX)

Epirubicin 50 mg/m^2^ IV on day 1, oxaliplatin 130 mg/m^2^ IV on day 1 with hydration, capecitabine 1,250 mg/m^2^/day PO in two divided doses continuously from days 1–21. Cycled every 21 days for 3 cycles preop and 3 cycles postop.

#### Fluorouracil + Leucovorin + Oxaliplatin (FOLFOX)

Day 1: oxaliplatin 85 mg/m^2^ IV infusion, 400 mg/m^2^ leucovorin IV infusion, followed by 5-FU 400 mg/m^2^ IV push then 5-FU 1,200 mg/m^2^ IV infusion for 22 h; day 2: 5-FU 1,200 mg/m^2^ IV infusion for 24 h daily on Days 1 and 2. Cycled every 14 days for 4 cycles. mFOLFOX6 is administered in cycles of 2 weeks for 4 cycles (= 8 weeks) on day 1, 15, 29 and 43. Oxaliplatin is given as a 2 h intravenous infusion at a dose of 85 mg/m2. Oxaliplatin needs to be diluted with 250–500 ml of 5% glucose solution and followed by leucovorin 400 mg/m^2^ iv over 2 h on day 1, and 5-FU 400 mg/m^2^ iv bolus on day 1, then 2,400 mg/m2 over 46–48 h continuous infusion.

#### Capecitabine + Oxaliplatin (CapeOx)

CapeOx is administered in cycles of 3 weeks for 3 cycles (= 9 weeks) on day 1, 22 and 43. Oxaliplatin is given as a 2 h intravenous infusion at a dose of 130 mg/m^2^ on day 1 followed by capecitabine given orally at a dose of 1,000 mg/m^2^ twice daily from the evening of day 1 to the morning of day 15 every 3 weeks.

#### Fluorouracil + Leucovorin + Oxaliplatin + Docetaxel (FLOT) (4)

FLOT is administered in cycles of 2 weeks for 4 cycles (= 8 weeks) on day 1, 15, 29, and 43 pre- and postoperatively. Docetaxel 50 mg/m^2^ is given as 1 h infusion, followed by oxaliplatin 85 mg/m^2^ as a 2 h infusion, leucovorin 200 mg/m^2^ over 2 h, and 5-FU 2,600 mg/m^2^ as a 24 h-infusion, with oral dexamethasone for prevention of fluid retention and allergic reactions. It is recommended to give an equivalent of 8 mg oral dexamethasone BID for 3 days starting from the day before docetaxel administration in the morning (8 a.m.) and evening (8 p.m.) on the day before administration of docetaxel, together with anti-emetic premedication on the day of chemotherapy and to continue 1 or 2 days after to minimize undesirable effects (Note: leucovorin may be replaced by sodium folinate according to local standards).

### Outcomes

#### Primary Endpoint

The primary endpoint in this study is Disease free survival (DFS).

This has been chosen because of the high proportion of high-risk patients who progress within a short timeframe after surgery. In the recent MRC ST03 trial which represents one of the most contemporaneous use of perioperative chemotherapy the 1-year DFS for node positive patients was only 60% with standard postoperative chemotherapy (completion of the perioperative concept) (28).

#### Secondary Endpoints

- Overall survival (OS).
- Pattern and rate of relapse.
- Rate of adverse events according to National Cancer Institute Common Terminology Criteria for Adverse Events (NCI-CTCAE v5.0).
- Global Health Status and Physical functioning according to EORTC QLQ C30 including nutritional status assessment.

#### Other Pre-specified Endpoints

- Quality of life according to EORTC QLQ C30 (and 6 additional items).
- Correlation of patient reported outcomes, quality of life, and nutritional status assessment with survival outcomes.

#### Screening Assessment

**Table:** 

**Participant timeline**			
	**Within 28 days (4 weeks) prior to randomization**	**Within 7 days prior to randomization**	**Within 1 day prior to randomization**
Medical history	X		
Adverse event assessment (CTCAE v5.0)		X	
Physical examination ^1^		X	
Performance status		X	
HBV, HCV, HIV serology		X	
Endocrine function^2^	X		
12-lead ECG (in triplicate) (2–5 min apart)	X		
LVEF (by MUGA or ECHO), only if indicated	X		
Disease assessment^3^	X		
Surgery assessment^¥^	X		
Review of prior/concomitant medications		X	
Quality of Life questionnaire (QLQ-C30)		X	
G8 screening for frailty for patients older than 70		X	
Hematology^4^		X^(A)^	
Serum chemistry^5^		X^(A)^	
Urine analysis		X^(A)^	
Pregnancy test^6^			X^(B)^
FFPE sample of biopsy	X		
FFPE sample of resection	X

X. In all cases 1: Including BP, pulse, temperature, weight, nutritional status.
2: Morning cortisol, ACTH, TSH (Free T3 and Free T4 in case of abnormal TSH).
3: Disease assessment: Full chest/abdomen/pelvis (CT and/or MRI); PET-CT scan may be an option.
¥Assessment of the resection of the primary tumor. Type of surgery and reconstruction performed (including if splenectomy was performed); resection margins (R0, R1, R2); Number of lymph nodes evaluated; Number of positive lymph nodes; Lymph node stations dissected based on the operative report; If applicable, type of the surgical complication graded according to the Clavien-Dindo Classification Version 2009 (29).
4: WBC, ANC, lymphocytes, eosinophils, basophils, RBC, hemoglobin, hematocrit, platelets; coagulation parameters.
5: Albumin, ALP, AST, ALT, GGT, LDH, Amylase, Serum phosphorous, Calcium total and ionized, Creatinine, Glucose, Lipase, Magnesium total and ionized, Potassium, Sodium, Total bilirubin, Uric acid; Calculated creatinine clearance; Direct and indirect bilirubin as clinically indicated if total bilirubin is ≥ 2xULN.
6: For pre-menopausal female subjects of childbearing potential only.
A: To be repeated if more than 14 days prior to treatment start.
B: Pregnancy test to be repeated if more than 24 h from treatment start.

#### Assessments During Treatment Period

**Table:** 

	**First 3 months after randomization (Months 1–3 = weeks 1–12)**		**Following 9 months (Months 4–12 = weeks 13–48?)**	
	**Every 2 or 3 weeks (+/– 5 days)**	**At 12 weeks (+/– 7 days)**	**Every 2 weeks (+/– 5 days)**	**Every 12 weeks (+/– 7 days)**
Adverse event assessment (CTCAE v5.0)	Both arms X	Both arms X	IO arm only X	Both arms
Disease assessment by imaging^3^		Both arms X		Both arms
Physical assessment	Both arms X	Both arms X	IO arm only X	Both arms
Nutritional assessment	Both arms X	Both arms X		IO arm only
Performance status	Both arms X	Both arms X	IO arm only X	Both arms
Quality of Life Questionnaire		Both arms		Both arms
12-lead ECG (in triplicate) (2–5 min apart); LVEF (by MUGA or ECHO)	Both arms, if clinically indicated			
Concomitant medications	Both arms X	Both arms X	IO arm only X	Both arms
Hematology^4^	Both arms X	Both arms X	IO arm only X	IO arm only
Serum chemistry^5^	Both arms X	Both arms X	IO arm only X	IO arm only
Thyroid function tests	IO Arm only,: every 6 weeks			
Cortisol levels^6^	IO arm only, every 6 weeks			
Pregnancy test^7^	Both arms, every 6 weeks		IO arm only, every 6 weeks	
Samples for biobanking/TR	Both arms X

X. Mandatory.
1: Including BP, pulse, temperature, weight.
3: Disease assessment: Full chest/abdomen CT and/or MRI.
4: WBC, ANC, lymphocytes, eosinophils, basophils, RBC, hemoglobin, hematocrit, platelets; coagulation parameters.
5: Albumin, ALP, AST, ALT, GGT, LDH, Amylase, Bicarbonate, Calcium total and ionized, Chloride, Creatinine, Glucose, Lipase, Magnesium total and ionized, Potassium, Sodium, Total bilirubin, Uric acid; Calculated creatinine clearance; Direct and indirect bilirubin as clinically indicated if total bilirubin is ≥ 2xULN.
6: Only if clinically indicated.
7: For pre-menopausal female subjects of childbearing potential only: urine or serum according to national regulations/institution guidelines.

### Assessment at End of Treatment for Both Arms

**Table:** 

	Normal completion* Both arms	**Treatment discontinuation Both arms**	
	**28 days after last treatment dose**	**Due to recurrence**	**Other reasons**
Adverse event assessment (CTCAE v5.0)	X	X	X
Clinical examination^1^	X	X	X
Hematology	X	X	X
Serum chemistry	X	X	X
Endocrine function tests	IO arm only	IO arm only	IO arm only
12-L ECG	X	X	X
Performance status	X	X	X
Disease assessment by imaging			X
Subsequent anti-cancer therapy^6^		X	X
Survival status	X	X	X
Quality of life	X according to specified timelines		X
Samples for biobanking and TR		X	According to specified timelines if patient is fit

X = mandatory.
*Control arm: completion of post-operative treatment according to the 2016 ESMO guidelines.
Experimental arm: completion of 1 year post-operative treatment with nivolumab 3 mg/kg IV Q2W plus ipilimumab 1 mg/kg IV Q6W for 1 year.

### Follow-up of Patients for Both Arms

**Table:** 

	**Follow up during Year 2 Both arms**	**Follow-up during Year 3 to 5 Both arms**
	**Every 3 months (± 7 days)**	**Every 6 months (± 14 days)**
Adverse event assessment (CTCAE v5.0)	X	X
Clinical examination^1^	X	X
WHO performance status	X	X
Disease assessment by imaging	X	X
Subsequent anti-cancer therapy^6^	X	X
Survival status	X	X
Quality of life	X at month 15	
Samples for biobanking and TR	X

X. In all cases.
1: according to standard of care and institution guidelines.
6: Each patient will be followed until death or for ~5 years following enrollment in order to document the long-term outcome (e.g., progression-free survival and overall survival).

### Sample Size

The primary objective of the study is to detect an increase in DFS with nivolumab plus ipilimumab given as adjuvant treatment. A phase III trial design with an increased one-sided type I error of 0.1 will be used for this Phase II trial.

The estimate for the control group is a DFS rate at 1 year of 65%. In ST03 (28), DFS rate at 1 year for node positive patients was 68% with 95% CI: 62–73% (subgroup analysis). In the MAGIC trial (7), in the 92 node positive patients who had surgery and postoperative chemotherapy, DFS (from surgery) rate at 1 year was 58% (95% CI: 47–67%).

The objective is to improve DFS rate at 1 year from 65 to 74% in the experimental arm. Assuming that DFS follows an exponential distribution, this corresponds to HR = 0.7 and an increase in median DFS from 19.3 to 27.6 months.

Using a one-sided log-rank test at a level of significance of 10%, a total of 142 events are required to reach 80% power.

Assuming an accrual rate at full speed (when all sites are open) of 10 patients per month and taking into account the opening of the sites, 2.52 patients/month are expected to be randomized for months 1–3, 4.13 patients/month for months 4–6, 5.80 patients/month for months 7–9, 7.56 patients/month for months 10–12 and 10 patients/month thereafter.

Taking into account an overall dropout rate of 5% at 1 year, we plan to randomize 240 patients in a 1:1 ratio between the control arm and the experimental arm in order to observe the required 142 events after an accrual period of 30 months and an additional follow-up of 22 months after closing the trial to patient entry. Total study duration is expected to be 52 months.

The rate of events in the control arm will be closely monitored during the trial to detect any departure from the assumption early on.

## Discussion

The VESTIGE trial is, to the best of our knowledge, the first to address switching adjuvant therapy in high risk patients with resected esophageal and gastric cancer following perioperative chemotherapy. It will offer patients the opportunity to either continue with the current standard of care (chemotherapy following surgery) or to be treated with combination immunotherapy with nivolumab and low dose ipilimumab. As the combination of nivolumab and low dose ipilimumab has been associated with promising response rates in patients with metastatic gastric and esophageal cancer, we hypothesize that this novel approach will be helpful in decreasing the risk of disease recurrence for high risk patients.

Historically, high dose ipilimumab with nivolumab has been associated with increased toxicity compared to nivolumab alone. Emergent data suggest that low dose ipilimumab with nivolumab has equivalent efficacy and reduced toxicity. For this reason, patients in VESTIGE will be treated with nivolumab and low dose ipilimumab. We also include the protocol management algorithms for most common toxicities associated with combination immunotherapy treatment.

Biomarkers associated with benefit from immunotherapy in gastric and esophageal cancer are under study, amongst these, PD-L1 immunohistochemistry and microsatellite instability are the most well-validated. In VESTIGE, we will incorporate a translational research programme including collection of pre-treatment biopsies, post-chemotherapy resection specimens and serial liquid biopsy on treatment to explore biomarkers predictive of immune checkpoint blockade efficacy.

In conclusion, VESTIGE is an international phase II randomized trial in high risk post resection gastric and esophageal cancers post neoadjuvant chemotherapy. The aim of the trial is to improve disease free survival by switching from standard of care adjuvant chemotherapy to combination immunotherapy with nivolumab and low dose ipilimumab. The trial opened to recruitment in July 2019 and will enroll 240 patients over a 2 year period. For enquiries please contact 1707@eortc.org.

## Data Availability Statement

All datasets for this study are included in the article/supplementary material.

## Ethics Statement

All patients will give informed consent and the trial will be conducted according to ICH-GCP and in accordance with the Declaration of Helsinki.

## Author Contributions

FL, ES, MN, MMa, AW, and MMo developed the concept. ES, MK, AG, MMa, MN, and NV developed the protocol. ES, AG, and FL wrote the manuscript. All authors reviewed the final manuscript.

### Conflict of Interest

The authors declare that the research was conducted in the absence of any commercial or financial relationships that could be construed as a potential conflict of interest.
